# Whole-genome sequencing reveals new insights into age-related hearing loss: cumulative effects, pleiotropy and the role of selection

**DOI:** 10.1038/s41431-018-0126-2

**Published:** 2018-04-30

**Authors:** Dragana Vuckovic, Massimo Mezzavilla, Massimiliano Cocca, Anna Morgan, Marco Brumat, Eulalia Catamo, Maria Pina Concas, Ginevra Biino, Annamaria Franzè, Umberto Ambrosetti, Mario Pirastu, Paolo Gasparini, Giorgia Girotto

**Affiliations:** 1 Medical Sciences, Chirurgical and Health Department, University of Trieste, Trieste, Italy; 2 Medical Genetics, Institute for Maternal and Child Health - IRCCS “Burlo Garofolo”, Trieste, Italy; 3 Institute of Molecular Genetics, National Research Council of Italy, Pavia, Italy; 4 Ceinge Advanced Biotechnology, Naples, Italy; 5 Neuroscience, Reproductive and Odontology Sciences Department, University of Naples “Federico II”, Naples, Italy; 6 UO Audiology, Fondazione IRCCS Ca Granda, Ospedale Maggiore Policlinico, Mangiagalli e Regina Elena, Milan, Italy; 7 Audiology Unit, Department of Clinical Sciences and Community Health, University of Milan, Milan, Italy; 8 Institute of Population Genetics, National Research Council of Italy, Sassari, Italy

## Abstract

Age-related hearing loss (ARHL) is the most common sensory disorder in the elderly. Although not directly life threatening, it contributes to loss of autonomy and is associated with anxiety, depression and cognitive decline. To search for genetic risk factors underlying ARHL, a large whole-genome sequencing (WGS) approach has been carried out in a cohort of 212 cases and controls, both older than 50 years to select genes characterized by a burden of variants specific to cases or controls. Accordingly, the total variation load per gene was compared and two groups were detected: 375 genes more variable in cases and 371 more variable in controls. In both cases, Gene Ontology analysis showed that the largest enrichment for biological processes (fold > 5, *p*-value = 0.042) was the “sensory perception of sound”, suggesting cumulative genetic effects were involved. Replication confirmed 141 genes, while additional analysis based on natural selection led to a prioritization of 21 genes. The majority of them (20 out of 21) showed positive expression in mouse cochlea cDNA and were associated with two functional pathways. Among them, two genes were previously associated with hearing (*CSMD1* and *PTRPD*) and re-sequenced in a large Italian cohort of ARHL patients (*N* = 389). Results led to the identification of six coding variants not detected in cases so far, suggesting a possible protective role, which requires investigation. In conclusion, we show that this multistep strategy (WGS, selection, expression, pathway analysis and targeted re-sequencing) can provide major insights into the molecular characterization of complex diseases such as ARHL.

## Introduction

Complex diseases are caused by several genetic and environmental factors, many of which still need further investigation [1]. Moreover, in contrast to monogenic disorders where causative genetic variants have near complete penetrance, complex diseases need specific approaches to be further investigated. The difficulty of exploring complex disease genetic models, lies in controlling the environmental components on one hand and accounting for different unknown genetic hypotheses on the other, such as pleiotropy, cumulative effects, gene–gene interactions etc. [2].

Age-related hearing loss (ARHL) or presbycusis is a very common complex disease leading to hearing deficits in the elderly. Due to the ageing population in our society, ARHL has reached to a 30% incidence among people over 50 years of age [3], becoming a major health care problem worldwide. ARHL significantly decreases the autonomy of affected people and also contributes to their anxiety, depression, and cognitive decline [4, 5]. The hearing loss is progressive, starting from high frequencies and subsequently affecting medium and lower frequencies as well. The cause is heterogeneous consisting of both environmental and genetic factors, and, although we have a reasonable understanding of the environmental risk factors, we know little about the genetic factors [6].

In particular, ARHL is characterized by an uncertain heritability and a higher genetic heterogeneity compared with other complex traits (i.e., diabetes, rheumatoid arthritis). Thus, it is not surprising that, up to now, there was a dearth of gene discovery despite some published Genome Wide Association  studies (GWAS) [7, 8]. Moreover, as genetic data become available on a large scale, pleiotropy increasingly becomes an interesting topic for complex and frequent diseases such as ARHL. Variability in complex diseases, such as ARHL, might be due to epigenetic changes during hair cell development and regeneration. To date, the possible involvement of epigenetic changes is still largely unknown and future studies are needed to shed light on this mechanism.

Recently, the possibility of analyzing whole-genome sequencing (WGS) and whole-exome sequencing (WES) data at population level has been highlighted [9]. However, it was shown that GWAS studies lose in power due to the allele frequency spectrum targeted by sequencing with realistic sample sizes [10]. Hence, it is necessary to explore new methodologies and approaches when dealing with WGS and WES data.

For the present work, WGS data for ARHL cases and controls from villages located in North-Eastern Italy (total of 156 subjects) were available, as well as individuals from an independent cohort of North-Western Italy, which was used for replication (56 subjects). Given the high complexity and genetic heterogeneity of the phenotype, we decided to explore a new scenario under the hypothesis that several variants contributing to the disease have a cumulative effect. To achieve this goal, we defined a pipeline characterized by the following steps: (a) identification of a burden of variants in WGS data significantly different among cases and controls, (b) natural selection analysis of genes in order to detect genes under selection, which can directly or indirectly influence the phenotype, (c) expression studies in the inner ear and (d) pathway analysis. Finally, to further validate our approach, target re-sequencing screening was carried out in an additional cohort of 389 ARHL patients for two genes previously reported as associated with hearing function in literature.

## Materials and methods

### Subjects

A WGS cohort of 378 individuals from the Friuli Venezia Giulia region in North-Eastern Italy was selected strictly for ARHL cases and controls based on the audiometric phenotype. A total of 156 subjects were considered for the discovery set: 78 cases and 78 controls matched by sex and age (mean age cases = 69, controls = 65; percentage of female cases = 60%, controls = 63%). Following the same selection criteria, a replication set of 56 subjects (28 cases and 28 controls) from an independent cohort located in North-Western Italy, Piemonte region (original cohort size *n* = 424) was enrolled, together with a completely independent cohort of 389 cases coming from several locations in Italy. Detailed demographic information about all cohorts is reported in Supplementary Table 1. Audiometric tests, a clinical examination and a lifestyle questionnaire were carried out for each subject. Considering that ARHL mainly affects hearing function at high frequencies, 4 and 8 kHz were measured for each subject and the average value was computed (pure tone average high (PTAH)). To avoid non-genetic variations in the hearing phenotype (e.g., monolateral hearing loss), the best hearing ear was considered for each individual. Subjects with history of occupational risk or pathologies related to ARHL were excluded from the study. Cases were defined as people >50 years old having PTAH ≥ 40, whereas controls were subjects >50 years old with PTAH ≤ 25. Blood samples were collected and DNA was extracted using a phenol–chloroform extraction procedure. Considering possible substructure in the genetic background of our samples, the overall genetic diversity (He) and inbreeding coefficient (Finbred) were estimated for cases and controls using PLINK v1.07 [11]. The Mann–Whitney test was used to evaluate the differences in these two statistics between the two groups.

### Whole-genome sequencing

Low coverage WGS data were generated using Illumina technology (Genome Analyzer and HiSeq 2000) at the Wellcome Trust Sanger Institute, Cambridge (UK) and Beijing Genomics Institute, Shenzhen (China). Data coverage ranged from 4× to 10×. Multi-sample genotype calling was performed using Samtools mpileup (v. 1.2) [12] and Variant Quality Score Recalibrator (VQSR) filtering was applied to the raw call data with GATK v.3.3 VariantRecalibrator module [13], separately for single nucleotide variants (SNVs) and insertions/deletions (INDELs). The filter creates a Gaussian Mixture Model by looking at annotation values over a high-quality subset of the input call set and then evaluates all input variants. The following parameters were used: for SNVs (I) Annotations: QD, DP, FS, HaplotypeScore, MQRankSum, ReadPosRankSum, InbreedingCoeff; (II) Training set: HapMap 3.3, Omni 2.5 M chip, 1000 Genomes Phase I; (III) Truth set: HapMap 3.3, Omni 2.5 M chip; (IV) Known set: dbSNP build 138; and for INDELs (a) Annotations: DP, FS, ReadPosRankSum, MQRankSum; (b) Training set: Mills-Devine, 1000 Genomes Phase I, dbSNP build 138; (c) Truth set: Mills-Devine; (d) Known set: Mills-Devine, dbSNP build 138. For each population, the lowest Variant Quality Score Log Odds Ratio (VQSLOD) threshold was determined by the output produced by VariantRecalibrator to select the best cut-off in terms of specificity and sensitivity of the trained model. For single nucleotide polymorphisms (SNPs), the selected minimum VQSLOD value was −15.0283 (99.80% truth sensitivity threshold). As INDEL calling and alignment is still more prone to error, a conservative approach was preferred, selecting a sensitivity threshold of 95%. The filter has been applied using GATK’s Apply Recalibration module.

To improve the quality of the raw genotyping results based on the low coverage sequencing data, several genotype refinement steps on the filtered call set were performed: (1) BEAGLEv4.r1230 [14] was used to assign posterior probabilities to all remaining genotypes. (2) SHAPEITv2 to phase all genotype calls [15] and (3) IMPUTEv2 [16] to perform internal imputation in order to correct for genotyping errors. Information about the ancestral allele and allele frequencies were retrieved from dbSNP build 138. The data are available through the European Genome/Phenome Archive (EGA) under the accession number EGAD00001002014 (https://www.ebi.ac.uk/ega/datasets/ EGAD00001002014).

### Data analysis

**Fig. 1:**
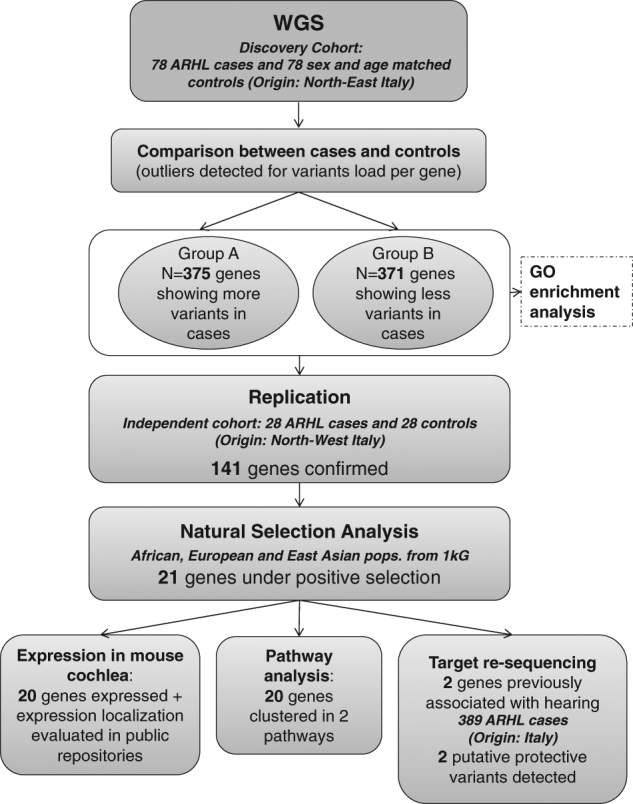
Workflow chart. The figure summarizes the workflow followed and described in the article

The complete workflow described below is summarized in Fig. 1.

#### Outlier genes analysis

Following the standard VCF file format, a variant was called if the alternative allele was present in at least one subject from each group (cases and controls). The definition of a gene was based on the Variant Effect Predictor v.74 [17] annotations and the number of variants was computed for each gene separately for cases and controls. The number of variants in controls was regressed with a linear regression against the number of variants in cases. Outlier genes were detected by selecting outlier residuals from the regression, defined as being >6 inter-quartile distances from the first and third quartile. For those genes, we separately analyzed the upper and lower outliers as being significantly more mutated in cases than in controls (Group A) and vice versa (Group B).

Statistical overrepresentation tests were carried out using the web tool PANTHER version 10.0 [18] with the following Gene Ontology (GO) and PANTHER annotation data sets: pathways, molecular function, biological process, cellular component and protein class. Bonferroni correction for multiple testing was applied. Results were compared with 100 random overrepresentation tests on the same number of randomly selected genes, in order to determine any false-positive recurrent enrichment.

The same outlier detection protocol was conducted in the replication cohort.

#### Variants classification and distribution in cases and controls

To further investigate which variants were accumulated in the outlier genes in cases and controls, respectively, we annotated all the variants using ANNOVAR [19]. For simplicity, we divided variants in “exonic” and “other”, grouping together the following annotations: intronic, upstream, downstream, UTR3, UTR5, non-coding RNA and splicing. We then compared variants present only in cases and only in controls vs. those that were shared.

#### Analyses of natural selection

The list of replicated genes was investigated for evidence of possible adaptation applying a selection scan based on PCA as described in Duforet-Frebourg et al. [20].

Considering that the analyzed cohorts were coming from Northern Italy villages, only SNPs that were differentiated along the European-Asian axis of variation (African, European and East Asian populations from 1000 Genome data) were considered in this study (cut-off a false discovery rate (FDR) of 0.1). Moreover, the derived allele frequency in the European and Asian population groups for each significant SNP was calculated.

For all the analyses, the functions implemented in the R package *“pcadapt”* have been used [21].

For each gene found under putative selection, we searched for any previous associations with other traits in the GWAS catalog [22] (available at: www.ebi.ac.uk/gwas. Accessed 30/08/2016), and we estimated the level of loss-of-function (LoF) intolerance (pLI) from the Exac database [23]. The closer pLI is to one, the higher the LoF intolerance, that is, a gene with pLI > = 0.9 could be considered as extremely LoF intolerant.

#### Pathway analysis

Genes under positive selection from the previous steps underwent identification of molecular network interactions and pathway analysis using the Ingenuity Pathway Analysis (IPA) tool by Ingenuity Systems (Redwood City, California, USA; http://www.ingenuity.com). Only direct interactions were taken into account and scores were computed for constructed networks based on the likelihood of the genes being connected together by random chance (significant threshold: score >2, with >99% confidence).

### Gene expression analysis

The expression of selected genes was tested in the inner ear of CD-1 mice. Total RNA was extracted from the whole cochleae of 2-month-old wild-type CD-1 mice using Direct-zol RNA MiniPrep Kit (Zymo Research). RNA was quantified using Nanodrop ND-1000 spectrophotometer (NanoDrop Technologies, Wilmington, DE, USA). Complementary DNAs were generated from 2 μg of total RNA using the Transcriptor First Strand cDNA Synthesis Kit (Roche) according to the manufacturer’s protocol. Complementary DNAs (cDNAs) were used for semiquantitative RT-PCR (sqRT-PCR). In all, 4 μl of cDNA were used for PCR amplification. Gene-specific primers spanning exon–exon boundaries were designed (primer sequence available upon request). β-Actin primers were used as internal control. PCR reactions were optimized to 95°C for 2 min, 30 amplification cycles at 95°C for 30 s, 60°C for 30 s, 72°C for 4 s and a final extension of 1 min at 72°C using Kapa HiFi HotStart ReadyMix PCR kit (Kapa Biosystem, Cape town, South Africa). Amplified products were resolved on 2% agarose gels and visualized by ethidium bromide staining.

### Targeted re-sequencing (TRS)

Two genes of interest were sequenced using the Ion Torrent PGM^TM^ (Life Technologies) platform. Genes were selected based on the results from the data analyses described. A total number of 389 ARHL cases were analyzed by TRS. Briefly, 10 ng of genomic DNA from whole peripheral blood was used to construct DNA libraries using the Ion AmpliSeq Library Kit 2.0 (Life Technologies, CA, USA), according to the manufacturer’s protocols. Template Ion Sphere Particles were prepared using the Ion PGM Template OT2 200 kit and a single end 200 base-read sequencing run was carried out using the Ion PGM sequencing 200 kit v2 (Life Technologies, CA, USA), on Ion Torrent PGM platform (Life Technologies, CA, USA). Sixty-four indexed patients’ libraries were sequenced simultaneously on each Ion 318 Chip. Sequencing data were then analyzed according to the Ion Torrent SuiteTM v3.6; SNVs and INDELs were collected into a standardized VCF version 4.1.

### Sanger sequencing

Specific variants of interest mentioned throughout the text were all confirmed by direct Sanger sequencing on a 3500 Dx Genetic Analyzer (Life Technologies, CA, USA), using ABI PRISM 3.1 Big Dye terminator chemistry (Life Technologies, CA, USA) according to the manufacturer’s instructions.

## Results

### Discovery set analysis

Cases and controls analyzed in our cohort were genetically homogeneous because no differences were found in genome-wide observed heterozygosity (Mann–Whitney *p*-value > 0.13) and inbreeding coefficient (Mann–Whitney* p*-value > 0.52).

**Fig. 2:**
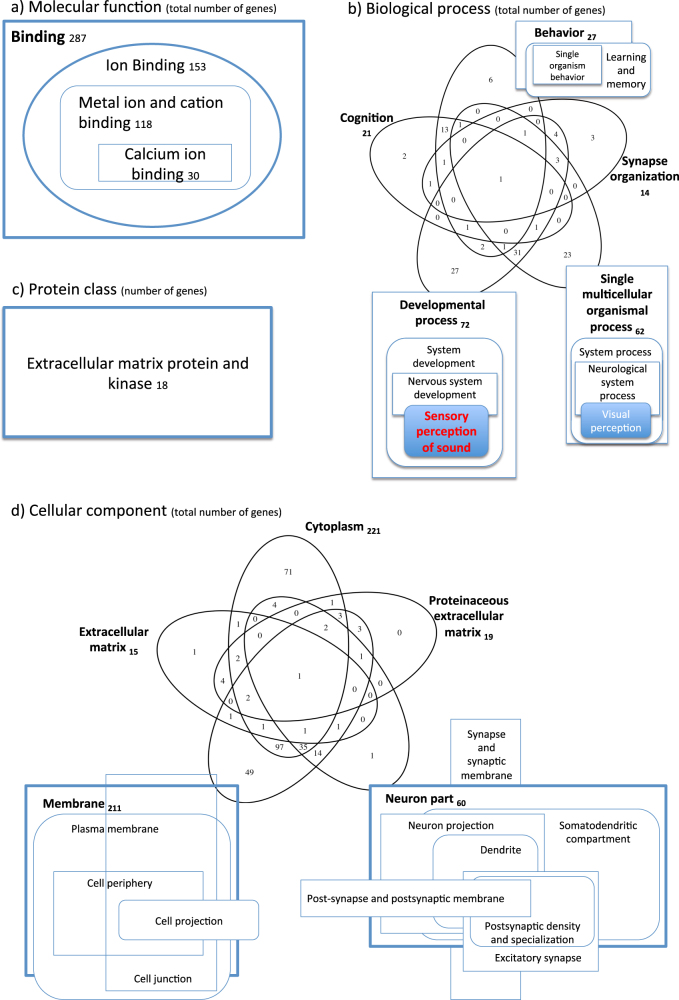
Enrichment analysis results. The figure displays enrichment analysis results for Group A genes, highlighting overlaps in terms of shared genes. The numbers indicate the number of genes listed for each feature. The following data sets were analyzed and are displayed separately: **a** molecular function; **b** biological process; **c** protein class; **d** cellular component

**Fig. 3:**
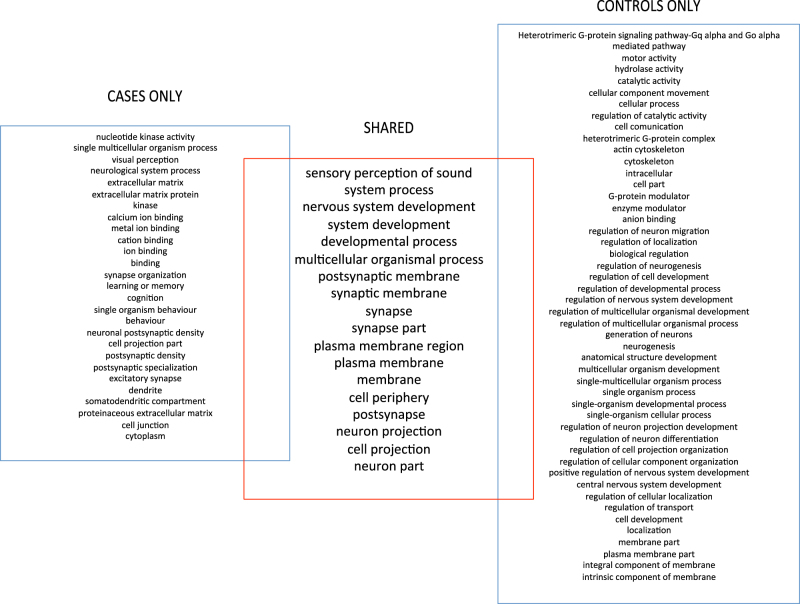
Overlap of enriched features between cases and controls. The figure summarizes all enriched features detected for Group A and Group B genes, showing which are in common between the two groups

In order to estimate differences in numbers of variants per gene between cases and controls, a linear regression analysis was performed. As reported in Table 1, the number of variants varied greatly among genes. The linear regression showed great consistency, with data following the diagonal line almost perfectly (regression line coefficient = 0.9938 and *R*^2^  =  0.9992). After selecting outliers using stringent criteria, a total of 746 genes were shortlisted with significantly more variants in cases than in controls (Group A, total of 375 genes) or significantly less variants in cases than controls (Group B, total of 371 genes). Complete lists of genes are reported in Supplementary Table 2. In Group A, overrepresentation tests of GO highlighted 45 significant biological/molecular processes for which our list of genes was enriched (Fig. 2). Regarding Group B, a total of 65 processes were significantly enriched, 18 of which were overlapping with those from Group A (Supplementary Table 3 and Fig. 3). Interestingly, in both groups, the “sensory perception of sound” biological function category was the most enriched among all categories (fold enrichment > 5).

**Table 1:** Summary information of variants detected in cases and controls

	Cases	Controls
Overall number of coding and non-coding variants	12,600,177	12,518,899
Median number of variants per gene	112	111
1st quartile (number of variants per gene)	49	49
3st quartile (number of variants per gene)	279	276
Mean number of variants per gene	281.1234	278.9673
Standard deviation	1003.006	997.2283

### Variants classification and distribution in cases and controls

**Fig. 4:**
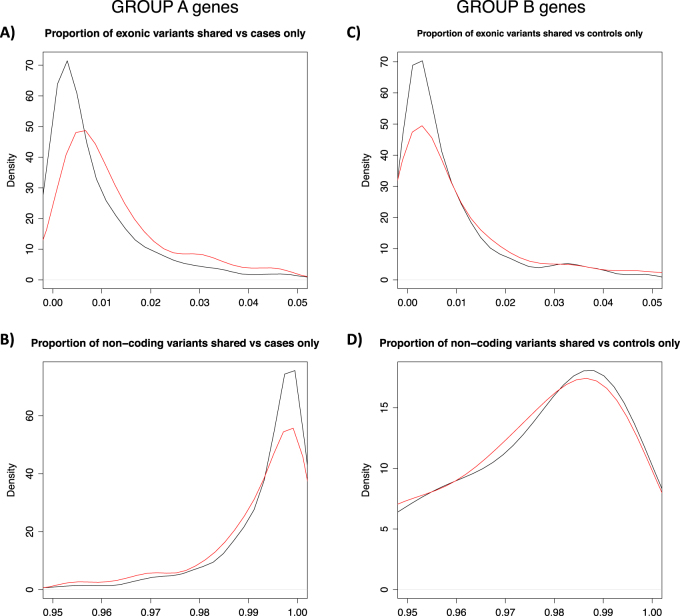
Distributions of coding and non-coding variants. The figure shows different distributions of coding and non-coding variants per gene. To overcome differences in gene size, only proportions of the total number of variants are shown: **a** Group A coding variants present in cases only (red line) compared with variants shared between cases and controls (black line); **b** Group A non-coding variants present in cases only (red line) compared with those shared between cases and controls (black line); **c** Group B coding variants present in cases only (red line) compared with those shared between cases and controls (black line); **d** Group B non-coding variants present in cases only (red line) compared with those shared between cases and controls (black line)

Among the 746 outlier genes, we compared the distributions of exonic (i.e., coding) variants with the non-coding ones. A Wilcoxon test showed a significant increase of the proportion of exonic variants detected in cases only compared with the proportion of exonic variants in the set of shared variants in Group A (*p*~2e-10, Fig. 4a), whereas the proportions of non-coding variants were equally distributed (*p* > 0.05, Fig. 4b). On the other hand, Group B genes were not enriched for exonic variants detected only in controls (Figs. 4c, d).

### Replication

The same protocol was used to replicate data in a completely independent cohort with another Italian geographic area (i.e., to overcome any confounding factors due to population structure or relatedness) [24]. Overall, 141 genes (57 out of 375 genes from Group A and 84 out of 371 from Group B) have been replicated (see Supplementary Table 2). In particular, 28,353 out of 149,798 variants detected in the 57 replicated genes in Group A were only present in cases. The vast majority of them (96 %) were intronic or intergenic variants, whereas the remaining 4% (1003 variants) were annotated as upstream, downstream, UTR3, UTR5, ncRNA or exonic variants (i.e., on average 7.5 alleles in each patient, and 6 alleles per gene). Regarding the 84 genes from Group B, 39,065 out of 224,384 variants were mainly intronic or intergenic (91%) and only present in controls. The remaining 9% of functionally relevant variants were present on average with 30 alleles per subject and 6 alleles per gene. Moreover, we found three genes with homozygous variants in cases only: *KCNIP4, MAST*, and *TIAM1*. Interestingly, the patient carrying the *TIAM1* homozygous variant is a 66 y.o. ARHL male showing a severe hearing loss at PTAH (102.5 dB). The variant is an exonic variant, rs34882418 (NM_003253.2:c.326C>T, NP_003244.2:p.(Thr109Ile)) predicted to be deleterious by SIFT, not present in the ClinVar database and extremely rare in the 1000 Genomes database (minor allele frequency = 0.005).

### Analyses of natural selection

Analysis of natural selection has been carried out to test if any of the 141 replicated genes could have been selected for having a major role on phenotypes directly or indirectly related to hearing. Moreover, we searched for evidence of possible adaptation along the European-East Asian axis. Twenty-one genes out of 141 (15%) were found to be under selection (13 from Group B and 8 from Group A) (see Table 2). Assessing LoF intolerance, a parameter to evaluate genes’ sensitivity to highly damaging variants, we identified two genes from Group A and seven genes from Group B that showed a probability of LoF intolerance (pLI) > 0.9, which means that they did not tolerate the presence of LoF alleles (see Table 2). Interestingly, all genes found to be under selection were also present in the GWAS catalog (i.e., already associated with a specific phenotype). One very promising candidate gene is *SDK1*, which has been already associated with vestibular phenotypes (i.e., motion sickness) and it also seemed to be very important for synapse formation/function and inner ear development (Table 2).

**Table 2:** Natural selection positive results with pLi and GWAS catalog associations for each gene

Gene	Group	SNPs with higher DAF in	Probability of LOF intolerance	GWAS catalog association
*ATP8A2*	B	Europe	0.00	Eating disorders, bipolar disorder schizophrenia
*FGF14*	B	East Asia	0.72	Preeclampsia, QT interval
*SEMA6D*	B	Europe, East Asia	1.00	Hair graying, Post bronchodilator FEV1/FVC ratio, BMI
*MYO1D*	B	Europe, East Asia	0.00	Refractive error
*SLC8A1*	B	Europe	0.99	QT interval
*SH3RF3*	B	Europe	0.98	facial and scalp hair features
*KCNH7*	B	Europe	0.98	Psoriasis, inflammatory skin disease
*PDE4D*	B	East Asia	0.98	Breast cancer, asthma, immune response to smallpox vaccine (IL-6), esophageal cancer
*GRIK2*	B	Europe, East Asia	0.99	Biochemical measures
*WBSCR17*	B	Europe	0.10	Response to montelukast in asthma (change in FEV1), response to angiotensin II receptor blocker therapy
*CSMD1*	B	Europe, East Asia	n.a	Menarche (age at onset), schizophrenia
*NRG1*	B	Europe, East Asia	0.95	Thyroid hormone levels, thyroid cancer, Hirschsprung disease
*PTPRD*	B	Europe, East Asia	1.00	Immune response to measles-mumps-rubella vaccine,T2D, restless legs syndrome
*ANTXR1*	A	Europe, East Asia	0.99	Height
*SDK1*	A	East Asia	0.01	Motion sickness, cognitive decline rate in late mild cognitive impairment, quantitative traits
*DGKI*	A	Europe	0.74	BMI, schizophrenia, cognitive decline (age-related), AIDS progression
*KIAA1217*	A	Europe	0.01	Cognitive performance, 3-hydroxy-1-methylpropylmercapturic acid levels in smokers
*DLG2*	A	East Asia	0.67	Mild influenza (H1N1) infection, Wilms tumor, influenza A (H1N1) infection
*WWOX*	A	Europe	0.00	Pulmonary function, 3-hydroxy-1-methylpropylmercapturic acid levels in smokers
*SNX29*	A	Europe	0.00	Visceral adipose tissue adjusted for BMI, schizophrenia, symmetrical dimethylarginine levels
*TIAM1*	A	East Asia	1.00	Hypertension, amyotrophic lateral sclerosis

*SNPs with higher DAF in* SNPs with higher derived allele frequency in, *Probability of LOF intolerance (pLi)* probability of loss-of-function intolerance: we consider pLI > = 0.9 as an extremely LoF intolerant set of genes, *GWAS catalog association* phenotype previously associated with the gene by means of gwas

### Pathway analysis of genes under selection

Finally, the 21 genes under selection have been investigated using IPA software in order to verify if they belong to any known pathway. Results showed two pathways, each including 10 focus molecules from our list of genes under selection. In particular, the first pathway (cell death and survival, nervous system development and function, tissue morphology, score = 24) contains the genes *ATP8A2* and *PDE4D* that have been already reported as expressed in the inner ear or involved in the hearing system [25, 26].

The second pathway (post-translational modification, cardiovascular system development and function, cell morphology, score = 24) contains, among others, four candidates *CSDM1, MYO1D, NRG1* and *PTPRD*, which have been previously detected to have a role in the hearing system and/or function [8, 27, 28].

### Expression analysis

**Fig. 5:**
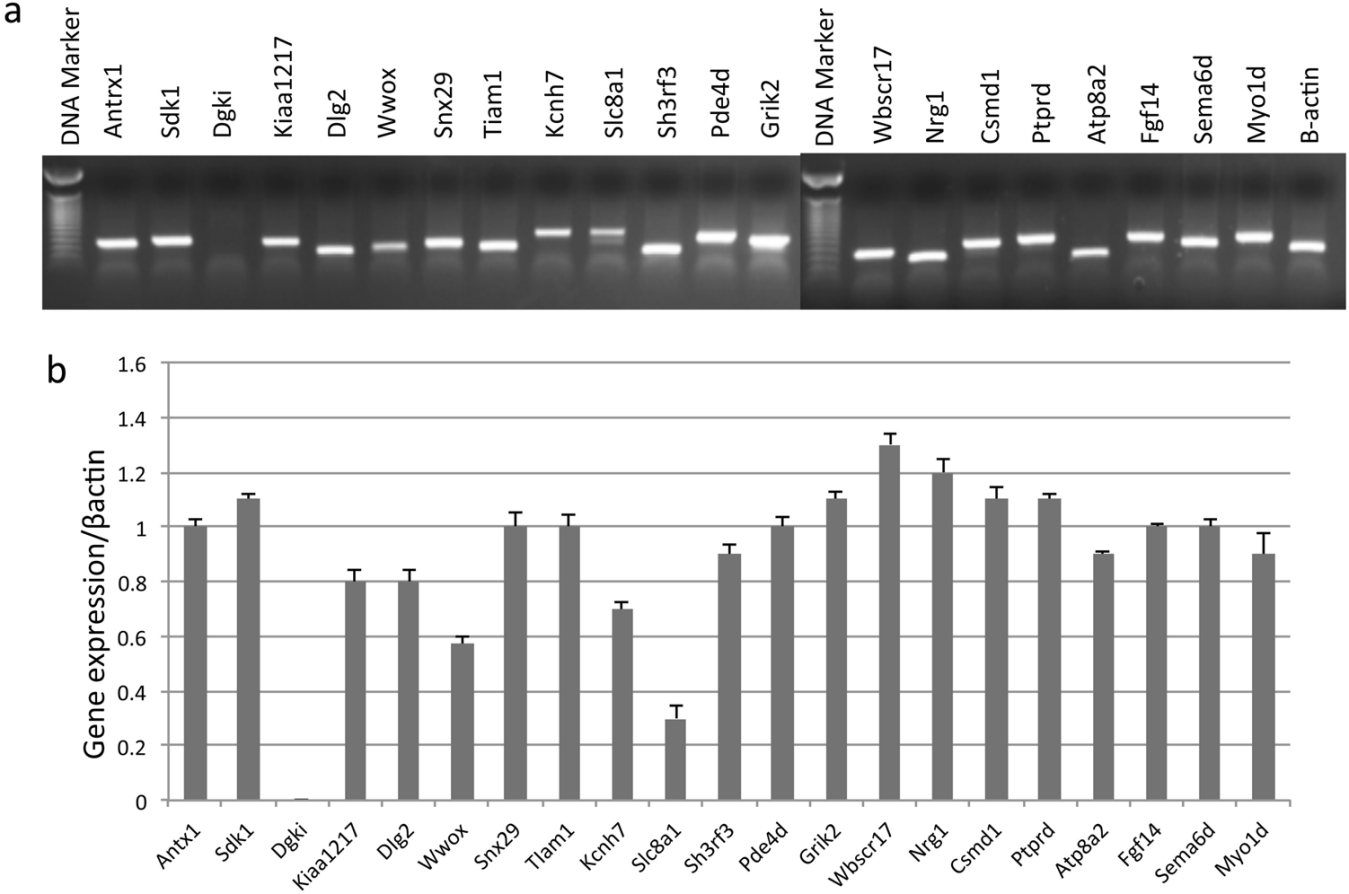
Expression profiles of genes under natural selection. The figure displays gene expression for 21 genes detected under natural selection, normalized to beta-actin: **a** sqRT-PCT on cDNA cochlea of adult mice; **b** agarose gel bands quantification. All genes, apart from one (*DGKI*) show good levels of expression

Expression analysis of the 21 strongly suggestive genes under selection was performed in the inner ear of 2-month-old mice. mRNA levels were normalized relative to the β-actin (Fig. 5). Interestingly, data analysis revealed positive expression for 20 genes (all but *Dgki*), suggesting a possible role in the auditory system. In order to broadly evaluate expression localization in the mouse inner ear, three publicly available resources were consulted: SHIELD, MGI and Gear (web resources). Results confirmed some level of expression for all the genes analyzed and all but three were expressed in hair cells of the cochlea. The *Dgki* gene, not detected in our experiments, was reported as weakly expressed in surrounding cells of the cochlea and of the vestibular system, as well as in auditory neurons. Detailed information is provided in Supplementary Table 4.

### TRS ARHL cohort

Two of the genes (*CSMD1* and *PTPRD*) from Group B and detected in the second IPA were previously described in two studies performed by our group, focusing on GWAS and replication in European and Asian samples. After statistical validation, both of them were investigated by immunocytochemistry in mouse cochlea and showed distinctive expression patterns [27] strongly suggesting a functional involvement in the auditory system. Based on this previous knowledge and experience in investigating these genes, we further prioritized them for performing target re-sequencing in a very large independent cohort of 389 ARHL cases. We focused on the 10 exonic variants (five for each gene) that we already detected only in controls in our discovery step. Six of them (three out of five for each gene, nonsynonymous and never reported in the 1000 Genomes and ESP databases) were not present in this large cohort (Table 3), thus suggesting a possible protective role to be further investigated.

**Table 3:** Targeted re-sequencing results for putative protective variants in *CSMD1* (NC_000008.10) and *PTPRD* (NC_000009.11), detected only in controls

Chr	Start	End	Ref	Alt	Gene	Exonic func.	AAChange	esp6500	1000g	snp138	AC contr WGS	Freq	AC target	Freq target
8	2,807,805	2,807,805	G	C	*CSMD1*	Nonsynonymous	CSMD1:NM_033225:exon67:c.10262C>G:p.(Ala3421Gly)	NA	NA	NA	1	0.0064	NA	NA
8	2,876,143	2,876,143	G	A	*CSMD1*	Nonsynonymous	CSMD1:NM_033225:exon52:c.7885C>T:p.(Pro2629Ser)	NA	NA	NA	2	0.0128	3	0.0032
8	3,008,942	3,008,942	C	G	*CSMD1*	Nonsynonymous	CSMD1:NM_033225:exon40:c.6008G>C:p.(Arg2003Thr)	NA	NA	NA	2	0.0128	NA	NA
8	3,165,321	3,165,321	A	T	*CSMD1*	Nonsynonymous	CSMD1:NM_033225:exon25:c.3846T>A:p.(His1282Gln)	NA	NA	NA	1	0.0064	NA	NA
8	3,263,571	3,263,571	G	A	*CSMD1*	Synonymous	CSMD1:NM_033225:exon15:c.2244C>T:p.(Ser748=)	0.0028	0.0028	rs146267457	4	0.0256	5	0.0054
9	8,465,660	8,465,660	A	T	*PTPRD*	Nonsynonymous	PTPRD:NM_001171025:exon14:c.2257T>A:p.(Ser753Thr),PTPRD:NM_001040712:exon15:c.2278T>A:p.(Ser760Thr),PTPRD:NM_130393:exon15:c.2272T>A:p.(Ser758Thr),PTPRD:NM_130391:exon16:c.2287T>A:p.(Ser763Thr),PTPRD:NM_130392:exon16:c.2287T>A:p.(Ser763Thr),PTPRD:NM_002839:exon32:c.3520T>A:p.(Ser1174Thr)	NA	NA	NA	1	0.0064	NA	NA
9	8,485,810	8,485,810	C	T	*PTPRD*	Nonsynonymous	PTPRD:NM_002839:exon28:c.3007G>A:p.(Gly1003Arg)	NA	NA	NA	1	0.0064	NA	NA
9	8,486,142	8,486,142	A	G	*PTPRD*	Nonsynonymous	PTPRD:NM_002839:exon28:c.2675T>C:p.(Val892Ala)	0.0031	0.001	rs151005956	1	0.0064	5	0.00543
9	8,486,278	8,486,278	C	A	*PTPRD*	Nonsynonymous	PTPRD:NM_002839:exon28:c.2539G>T:p.(Val847Leu)	0.0002	NA	rs143787300	2	0.0128	2	0.00217
9	8,492,901	8,492,901	C	A	*PTPRD*	Nonsynonymous	PTPRD:NM_002839:exon27:c.2428G>T:p.(Ala810Ser)	NA	NA	NA	1	0.0064	NA	NA

*Chr* chromosome, *Start* start position,* End* end position, *Ref* reference allele, *Alt* alternative allele, *Gene* gene name,* Exonic func.* exonic function, *AAChange* amino-acid change, *esp6500* frequency reported in the Esp6500 database, NA if variant is not reported, *1000g* frequency reported in 1000 Genomes database, NA if variant is not reported, *snp138* rs code if available, NA if rs code is not available, *AC contr WGS* allele count in the discovery cohort, *Freq* frequency in the discovery cohort, *AC target* allele count in the target sequencing cohort of 389 cases, NA if variant was not reported, *Freq. target* frequency in the target sequencing cohort of 389 cases, NA if variant was not reported

## Discussion

ARHL is a complex and heterogeneous disorder. Despite all the efforts made in gene discovery, little is still known about the genetic risk factors underlying the disease itself. In this light, identifying cumulative and polygenic effects involved in this disease is a big challenge. In this study, we conducted a WGS analysis in a large cohort of ARHL cases and healthy controls to identify genes and variants involved in ARHL. In particular, we showed that the strength of our approach relies on a novel strategy to query WGS data that is not based on association analysis. This strategy allowed us to identify genes enriched in several relevant biological and molecular processes such as “sensory perception of sound” (i.e., strictly connected with the phenotype investigated) or “neurological system development”. These findings might play an important role in ARHL and hearing function, considering that the hearing system is connected to the brain via the cochlear nerve, and most of the hearing genes are expressed in the brain [29]. Given that genes were selected for a differential burden of variants between the two groups (cases or controls), the presence of functional enrichment is evidence of polygenic and cumulative effects involved in ARHL. Accordingly, we demonstrated that case-specific exonic variants were significantly enriched, further suggesting that an accumulation of coding variants might contribute to the pathogenicity of a complex disease such as ARHL. Of course in the future, attention should be paid to regulatory elements, which might also contribute to the phenotype similar to what has been described for other complex diseases [30].

Considering the difficulties underlying genetic replication studies (e.g., due to different population structure), the high replication rate of our strategy (i.e.,19%) further confirms its validity. The majority of replicated genes do not seem to be under natural selection, which means they accumulated variants in a neutral or relaxed fashion. This is in line with other genes associated with complex traits, which display an excess of negative selection [31]. This finding is also consistent with data showing that the hearing phenotype and related genes, such as Connexins, are affected by both assortative mating and relaxed purifying selection [32–34]. Nevertheless, a subset of these genes (21 out of 141) was estimated to be under natural selection. They were already present in the GWAS catalog, thus being associated with other phenotypes (i.e., not hearing) suggesting the presence of pleiotropy. Expression studies revealed that most of these genes were expressed in the inner ear.

The usefulness of our approach is further corroborated by the following examples. First, genes under selection in Group A should likely be those having stronger effects. Accordingly, a predicted deleterious homozygous variant in *TIAM1* gene was detected in a patient, whose audiometric phenotype was the severest among all cases investigated. Most likely, the presence of this allele might be enough to explain the phenotype in this patient resembling a monogenic form of hearing loss. This agrees with recent results showing a continuum of effect sizes ranging from extremely rare variants driving Mendelian disorders to common variants with small effects involved in complex forms of the phenotype [35]. Moreover, *TIAM1* is involved in regulating tissue polarity of hair cells by interacting with the *LIS1* gene [36] and it also shows a high LoF intolerance (pLI = 1) further suggesting a possible pathogenic role.

The second example is related to genes under selection from Group B. Two of them, CSMD1 [37, 38] and PTPRD [39], were previously associated with other phenotypes. More recently, they were associated with the hearing function [8, 27] and replicated in a trans-ethnic cohort. Expression profiles in mouse cochlea by immunocytochemistry showed striking patterns suggesting a role in hearing function [27]. Building on this previous knowledge, a TRS study in a series of ARHL patients is reported identifying six variants with a possible protective role for ARHL and confirming the pleiotropic effect of genes under selection.

Regarding genotype–phenotype correlation, further functional experiments (i.e., immunohistochemistry and confocal experiments) to determine the exact localization and the role of the gene/proteins identified will possibly lead to a specific correlation between the audiometric profile and the relative gene. All these results highlight the complexity of the hearing phenotype, its polygenic nature, and the likely effects of genetic variation in the affected patients.

## Conclusions

Following the hypothesis of cumulative effects of several variants contributing to the disease, we identified a list of candidate genes for ARHL by applying a new strategy based on different complementary approaches using WGS data. In conclusion, this study provides a list of 21 candidate genes, identified due to a differential mutational burden in ARHL cases and controls, with evidence of natural selection and expression in mouse cochlea. The molecular screening of these candidates in larger cohorts of cases and controls could be included in future NGS approaches for the identification of genetic risk factors and their individual contribution to the etiology of ARHL and to further understand how they interact with each other. Moreover, strong evidence of a possible role in the disease was obtained for two genes (i.e., *CSMD1* and *PTPRD*) and a series of variants detected in their sequences. These findings underline the need for additional functional studies of both *CSMD1* and *PTPRD* genes/variants (e.g., in animal models) to definitively prove their role in ARHL. Identifying genetics risk factors for ARHL and improving appropriate interventions will be a worthwhile goal for future research and results presented in this article will be a valuable starting point.

## Web resources

MGI: http://www.informatics.jax.org/ [accessed November 2017];

SHIELD: https://shield.hms.harvard.edu/ [accessed November 2017];

g-EAR: http://gear.igs.umaryland.edu/ [accessed November 2017].

## Electronic supplementary material

Supplementary_Table_1

Supplementary_Table_2

Supplementary_Table_3

Supplementary_Table_4
